# Introduction to carbon nanomaterials for smart applications

**DOI:** 10.1039/d5na90013a

**Published:** 2025-02-21

**Authors:** Zhenyuan Xia, Yeye Wen, Muqiang Jian

**Affiliations:** a Chalmers University of Technology Sweden; b Beijing Institute of Technology China; c Beijing Graphene Institute China

## Abstract

Zhenyuan Xia, Yeye Wen and Muqiang Jian introduce the *Nanoscale Advances* themed issue on carbon nanomaterials for smart applications.
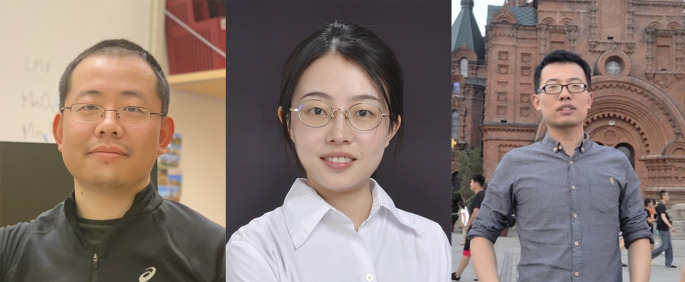

Carbon is often regarded as a “magic” element due to its unparalleled versatility in hybridization, enabling the formation of a diverse array of carbon-based materials. Among these, carbon nanomaterials stand out as a fascinating class, emerging alongside the rapid advancements in nanotechnology. Carbon nanomaterials span a range of dimensionalities, from zero-dimensional fullerenes to one-dimensional carbon nanotubes (CNTs), two-dimensional graphene and graphdiyne, and three-dimensional nanodiamonds. Their structures, dictated by the hybridization states of carbon atoms and their nanoscale geometries, bestow them with remarkable physical and chemical properties. For instance, fullerenes exhibit exceptional antioxidant capabilities, while CNTs and graphene demonstrate extraordinary mechanical properties of ultrahigh tensile strength in the range 100–130 GPa and Young's modulus exceeding 1 TPa, and electrical properties of about 10^6^–10^7^ S m^−1^ for CNT and 10^8^ S m^−1^ for graphene. These exceptional properties position carbon nanomaterials as pivotal players in the development of next-generation technologies.

For decades, tremendous efforts have been directed towards achieving precise control over the preparation and fabrication of carbon nanomaterials, a pursuit that has profoundly influenced the field of materials science. Distinguished by their unique optical, electrical, thermal, and mechanical properties, carbon nanomaterials have proven to be indispensable components in the development of smart materials. These advancements present unparalleled opportunities to tackle pressing challenges across a broad spectrum of applications, including energy conversion and storage, environmental sustainability, and advanced electronics.

In this themed collection of *Nanoscale Advances*, we aim to provide a forum that broadly focuses on the preparation of carbon nanomaterials and their diverse smart applications. The selection includes both review and research articles, offering insights into the design, synthesis and functionalization of carbon nanomaterials, as well as their assembly for smart applications. Applications cover wearable electronics, structural materials, water treatment, and energy conversion and storage devices, showcasing their exceptional potential in shaping next-generation technologies. Zhang *et al.* (https://doi.org/10.1039/D4NA00437J) provide an insightful review on the controlled synthesis and applications of ultralong CNTs. These defect-free, high-purity CNTs exhibit extraordinary mechanical, electrical, and thermal properties, making them ideal for applications such as super-strong fibers, smart sensors and electronic devices. Gao *et al.* (https://doi.org/10.1039/D4NA00701H) explore the recent advances in microwave-assisted synthesis techniques for carbon-based materials. These methods leverage the unique interactions of microwaves with carbonaceous precursors, enabling scalable synthesis of carbon nanomaterials while minimizing energy consumption. Such approaches represent a paradigm shift in the sustainable fabrication of carbon nanostructures. Kovtun *et al.* (https://doi.org/10.1039/D4NA00359D) report a novel method for covalent functionalization of graphene through blue-light-activated radicals. This precise approach to modifying graphene's structure highlights its versatility for smart applications in electronics, energy storage, and sensing technologies. Dryfe *et al.* (https://doi.org/10.1039/D4NA00506F) explore the wetting properties of carbon surfaces, including glassy carbon and graphite substrates. The result has important reference value for the smart application of these materials in the field of electrochemistry.

This themed collection further demonstrates the integration of super-aligned carbon nanotube films with thermal-resistant zirconia fibers to develop lightweight lightning strike protection composites (https://doi.org/10.1039/D4NA00392F). These advanced composites demonstrate enhanced electrical conductivity, mechanical robustness, and notable weight reduction, making them promising candidates for applications in aerospace engineering. This study underscores the multifunctional capabilities of CNT-based materials under extreme operating conditions. Environmental sustainability is another theme of this collection. Galiotis *et al.* (https://doi.org/10.1039/D4RA05658B) introduce graphene oxide and nanoplatelet hybrid aerogels designed for the removal of emerging contaminants from water. These aerogels exhibit superior adsorption properties, providing a scalable and sustainable solution for water purification and addressing global concerns about water safety. Carbon nanomaterials also play a critical role in revolutionizing energy conversion and storage technologies. Ciesielski *et al.* (https://doi.org/10.1039/D4NA00600C) report the synthesis of a manganese-iron dual metal–organic framework (MOF) cathode material, enhanced with carbon-based components, that achieves remarkable capacity and stability in lithium metal batteries over extended cycles. Similarly, Shao *et al.* (https://doi.org/10.1039/D4NA00569D) provide a comprehensive review of carbon-nanomaterial-assisted fibrous zinc-ion batteries. These batteries exhibit excellent mechanical flexibility and electrochemical performance, highlighting the pivotal role of carbon nanomaterials in developing high-performance, sustainable energy storage systems.

This collection exemplifies the remarkable versatility of carbon nanomaterials, showcasing their application across a diverse spectrum of fields, including flexible electronics, wearable energy systems, environmental sustainability, and aerospace innovations. The advancements and insights presented within these studies provide a foundation for future research, highlighting the critical importance of continued exploration into the preparation, assembly, and integration of carbon nanomaterials.

As Guest Editors of this themed issue, we wish to express our heartfelt gratitude to *Nanoscale Advances* for offering this platform to researchers from diverse disciplines, fostering the exchange of ideas and advancements in the smart application of carbon nanomaterials. We are especially grateful to the editorial team of *Nanoscale Advances* for their invaluable support, with particular thanks to Dr Zifei Lu for her guidance and assistance throughout the preparation of this issue. Our sincere appreciation extends to all the authors for their outstanding contributions and to the reviewers for their expert evaluations, which have been instrumental in shaping the quality and impact of this collection.

